# A machine learning model for predicting anatomical response to Anti-VEGF therapy in diabetic macular edema

**DOI:** 10.3389/fcell.2025.1603958

**Published:** 2025-05-30

**Authors:** Wenrui Lu, Kunhong Xiao, Xuemei Zhang, Yuqing Wang, Wenbin Chen, Xierong Wang, Yunxi Ye, Yan Lou, Li Li

**Affiliations:** ^1^ Shengli Clinical Medical College of Fujian Medical University, Fujian Provincial Hospital, Fuzhou University Affiliated Provincial Hospital, Fuzhou, China; ^2^ Department of Ophthalmology and Optometry, Fujian Medical University, Fuzhou, China; ^3^ School of Basic Medical Sciences, Fujian Medical University, Fuzhou, China; ^4^ Department of Computer, School of Intelligent Medicine, China Medical University, Shenyang, China

**Keywords:** diabetic macular edema, anti-vegf, predictive model, nomogram, diabetic retinopathy

## Abstract

**Purpose:**

To develop a machine learning model to predict anatomical response to anti-VEGF therapy in patients with diabetic macular edema (DME).

**Methods:**

This retrospective study included patients with DME who underwent intravitreal anti-VEGF treatment between January 2023 and February 2025. Baseline data included optical coherence tomography (OCT) features and blood-based metabolic and hematologic markers. The primary outcome was defined as a ≥20% reduction in central retinal thickness (CRT) post-treatment. Feature selection was performed using univariate logistic regression and LASSO regression. Five machine learning algorithms—logistic regression, decision tree, multilayer perceptron, random forest, and support vector machine—were trained and validated. Model performance was evaluated using accuracy, sensitivity, specificity, Area Under the Receiver Operating Characteristic Curve (AUC), and decision curve analysis. The best-performing model was further interpreted using SHAP analysis, and a nomogram was constructed for clinical application.

**Results:**

Among the 37 baseline variables, five key predictors were identified: preoperative CRT >400 μm, presence of retinal edema, presence of subretinal fluid (SRF), disorganization of the inner retinal layers (DRIL), and ellipsoid zone (EZ) integrity. The logistic regression model achieved the best performance with an accuracy of 0.83, sensitivity of 0.85, specificity of 0.79, and an AUC of 0.90 (95% CI: 0.81–0.99). SHAP analysis revealed that preoperative retinal edema, DRIL, SRF, and CRT had the strongest positive contributions, while intact EZ was a negative predictor of CRT reduction. A nomogram was developed to facilitate individualized clinical decision-making.

**Conclusion:**

We successfully developed a predictive model for anatomical response to anti-VEGF therapy in DME patients. The model identified key features associated with treatment outcomes, providing a valuable tool for personalized therapeutic planning. Further validation in multicenter cohorts is warranted to confirm generalizability and enhance model robustness.

## 1 Introduction

Diabetic macular edema (DME) is the most common vision-threatening complication arising from diabetic retinopathy (DR).Globally, approximately 5.5% of individuals with diabetes are affected by DME (Im et al., 2022).Intravitreal injection of anti-vascular endothelial growth factor (anti-VEGF) agents has become the first-line treatment for DME owing to their potent anti-permeability and anti-angiogenic effects (Ehlers et al., 2022). However, clinical practice reveals significant inter-individual variability in response to anti-VEGF therapy. Approximately 30%–40% of patients continue to exhibit persistent edema or suboptimal visual improvement despite regular treatment (Zhang et al., 2022; Hussain and Ciulla, 2016). These patients often require additional therapeutic interventions such as laser photocoagulation, corticosteroid injections, or even surgical treatment (Hussain and Ciulla, 2016; Hatamnejad et al., 2024). Therefore, early identification of high-risk individuals who are likely to have poor treatment responses and the development of individualized intervention strategies have become critical challenges in current DME management.

In recent years, artificial intelligence (AI) has shown great promise in the management of chronic ocular conditions, including dry eye disease (Li et al., 2024; Wei et al., 2024), myopia (Xiao et al., 2024), and age-related macular degeneration (AMD) (Waldstein et al., 2020; Gong et al., 2024; Wan et al., 2025). In the field of DR, AI applications have primarily focused on early screening and diagnosis. AI systems have demonstrated high efficiency and accuracy in DR screening, enabling automated analysis of fundus images without human intervention to detect referable DR and DME. These systems have achieved sensitivity and specificity comparable to, or even exceeding, those of human graders in certain settings (Bellemo et al., 2019; Farahat et al., 2024; Yuan, 2019; Guo et al., 2023). However, compared to screening and diagnosis, the application of AI in predicting treatment response to anti-VEGF therapy in DME remains in its early stages of exploration.

Based on this, this study aims to develop a predictive model by collecting pre-treatment ocular parameters and blood-based biomarkers as predictor variables, with post-treatment resolution of macular edema as the outcome variable. This model is expected to assist clinicians in identifying potential non-responders at the initial stage of diagnosis and treatment planning, allowing for more targeted and personalized intervention strategies.

To our knowledge, this is the first study to integrate pre-treatment structural OCT features with a comprehensive panel of systemic blood biomarkers to predict response to anti-VEGF therapy in DME patients. This approach extends beyond previous models that relied solely on imaging data by capturing systemic factors that may influence treatment outcomes (Meng et al., 2024; Mao et al., 2022). In addition, by incorporating SHAP analysis and nomogram visualization, our model offers enhanced interpretability for the prediction of treatment response, providing insights into the contribution of each feature and distinguishing our approach from prior black-box machine learning methods.

## 2 Methods

### 2.1 Study population and data collection

This study was conducted at Fujian Provincial Hospital from January 2023 to February 2025. Patients diagnosed with DME and initial intravitreal with anti-VEGF were consecutively enrolled. All included patients had complete preoperative optical coherence tomography (OCT) results and blood biochemical data. Baseline center involved DME (SD-OCT central subfield thickness [CST], ≥250 μm). Exclusion criteria included: prior history of anti-VEGF treatment, presence of severe ocular conditions that could affect retinal thickness evaluation, cataract surgery within 6 months before baseline, comorbid systemic diseases that might interfere with study variables, or incomplete clinical data for any reason. All patients received intravitreal anti-VEGF injections administered by the same experienced ophthalmologist to ensure consistency in treatment protocol. The study was approved by the Ethics Committee of Fujian Provincial Hospital (K2025-03-064) and strictly adhered to the principles of the Declaration of Helsinki. Written informed consent was obtained from all participants.

To assess whether the available sample size was adequate for developing a reliable prediction model, we performed a minimum sample size calculation using the pmsampsize package (Ensor et al., 2019). Assuming a binary outcome with an expected prevalence of 30%, a target c-statistic of 0.80, and five predictor parameters, the required sample size was estimated to be 172 participants with approximately 52 events, based on Criterion 1 (which ensures a shrinkage factor ≥0.90 to reduce overfitting).

Collected demographic and clinical information included sex, age, baseline best-corrected visual acuity (BCVA), history of hypertension, diabetes duration (>10 years or not), and whether insulin therapy was being used. A 1-mm-wide area centered on the foveal depression was analyzed for each B-scan using a standard template. OCT imaging parameters included baseline retinal edema thickness >400 μm (yes/no), central retinal thickness (CRT), presence of subretinal fluid, integrity of the external limiting membrane (ELM), ellipsoid zone (EZ), and retinal pigment epithelium (RPE), as well as disorganization of the inner layers (DRIL) and outer retinal layers. These specific OCT features were selected due to their reported relevance in predicting DME treatment outcomes, as they reflect key aspects of retinal structure and edema known to affect prognosis (Sen et al., 2025; Nakano et al., 2019).

Blood biochemical markers included: total bilirubin, triglycerides, total cholesterol, high-density lipoprotein (HDL), low-density lipoprotein (LDL), apolipoprotein A, fasting blood glucose, urea, creatinine, uric acid, calcium, and magnesium. Hematological parameters included: white blood cell count, neutrophil count, lymphocyte count, red blood cell count, hemoglobin, platelet count, mean platelet volume (MPV), and platelet distribution width (PDW).Diabetes-related markers included: glycated hemoglobin in mmol/L (HbA1c) and percentage (HbA1c, %). These systemic biomarkers were chosen to represent metabolic control, renal function, and inflammatory status, factors that are implicated in diabetic microvascular complications like DME (Sen et al., 2025; Zhou et al., 2022).

**FIGURE 1:**
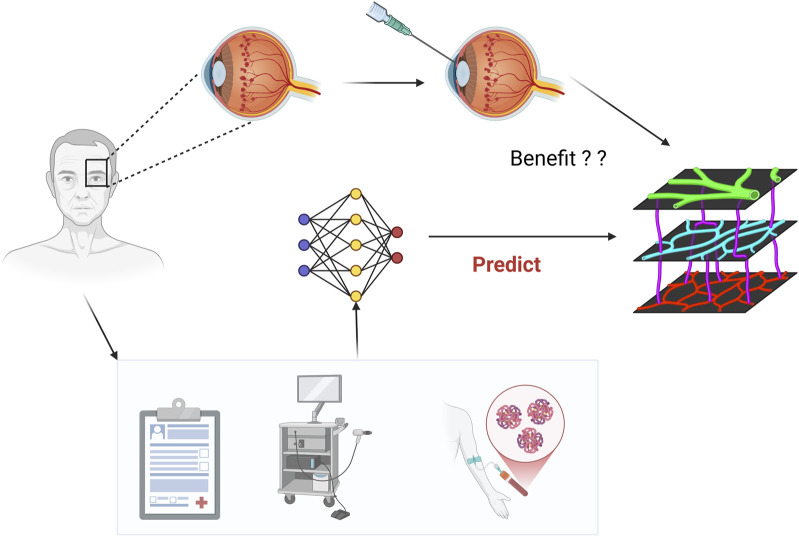
Workflow for predicting anatomical response to Anti-VEGF therapy in DME.

The primary outcome variable was defined as a ≥20% reduction in central retinal thickness post-treatment, based on OCT measurements, to evaluate the short-term anatomical response to anti-VEGF therapy. The overall study workflow is illustrated in Figure 1.

### 2.2 Feature selection

To identify candidate variables for the predictive model, univariate logistic regression analysis was performed to screen for potential factors associated with post-treatment central retinal thickness reduction. To further eliminate redundant variables and identify those with the greatest predictive influence, we employed Least Absolute Shrinkage and Selection Operator (LASSO) regression with L1 regularization for feature selection.

LASSO applies a penalty to regression coefficients, shrinking some to zero and thereby retaining only the most relevant predictors. We used 10-fold cross-validated LASSO (via the cv.glmnet function) to select the optimal regularization parameter (λ). The final λ value was determined using the 1-standard error (1-SE) rule (lambda.1se), and variables corresponding to non-zero coefficients were selected as candidate predictors. The final features included in the machine learning models were determined by combining variables identified by both univariate logistic regression and LASSO regression.

### 2.3 Model development and evaluation

Several machine learning algorithms were used to build predictive models, including decision tree (DT), logistic regression, multilayer perceptron (MLP), random forest (RF), and support vector machine (SVM). The dataset was randomly split into a training cohort (80%) and an internal validation cohort (20%).

During model training, each algorithm underwent hyperparameter optimization using 10-fold cross-validation and grid search to enhance performance. To evaluate the effectiveness of each model in predicting macular edema resolution after anti-VEGF treatment, we compared their performance on the validation set using the following metrics: Accuracy, Sensitivity, Specificity, Receiver Operating Characteristic (ROC) curves, Decision Curve Analysis (DCA).

The model with the best overall performance was selected for further analysis. Based on this optimal model, we developed a prediction tool, and conducted feature importance analysis and Shapley Additive Explanations (SHAP) to enhance model interpretability and transparency.

### 2.4 Statistical analysis

All continuous variables were first tested for normality using the Shapiro–Wilk test. As the test results indicated that all variables had P values less than 0.05 (Supplementary Table S1), the data were considered non-normally distributed. Therefore, continuous variables were summarized using medians with interquartile ranges (IQR: P25, P75), and intergroup comparisons were performed using the Mann–Whitney U test. Categorical variables were presented as frequencies and percentages (%), and compared using the chi-square test.

All 37 candidate features had a missing rate of 0%, and therefore no imputation was required. Outlier detection did not reveal any observations that needed to be excluded.

Several continuous variables were converted into categorical variables based on clinically established thresholds from previous literature and guidelines (e.g., CRT >400 μm, HbA1c ≥ 8%). This step helped reduce scale discrepancies while preserving clinical interpretability. All binary features were encoded as 0 and 1.

Model construction was carried out using the following functions and engines from the tidymodels framework. To enhance the interpretability of the model, SHAP values were computed to quantify the contribution of each feature to the prediction outcome. SHAP analysis was performed using the shapviz package. A higher SHAP value indicates a greater influence of that feature on the prediction, while positive and negative values represent positive or negative impacts, respectively. SHAP summary plots and dependence plots were generated to identify the most influential variables.

To improve the clinical applicability of the model, a nomogram was developed based on the logistic regression model. The nomogram was constructed using the nomogram () function in the rms package. A logistic regression model was fitted using lrm (), and the significant variables were then visualized to generate individualized risk scores, providing a practical tool for personalized prediction in clinical settings. All statistical analyses were conducted using R software version 4.4.1.

## 3 Results

### 3.1 Patient characteristics

Significant differences in baseline characteristics were observed between patients who experienced postoperative central retinal thickness reduction and those who did not (Table 1). Among patients with a reduction in retinal thickness, the proportion of individuals with preoperative retinal edema >400 μm and the presence of subretinal fluid was significantly higher (*p* < 0.001). Regarding retinal structure, the integrity of the ELM and the EZ was significantly more common in patients who showed a reduction in retinal thickness (*p* < 0.001).

**TABLE 1:** Comparison of baseline characteristics between patients with and without retinal thickness decrease.

Variable	Non ≥20% CRT reduction N = 133^a^	≥20% CRT reduction N = 71^a^	p-value^b^
Gender			0.6
Male	77 (58%)	44 (62%)	
Female	56 (42%)	27 (38%)	
Hypertension			>0.9
Not	80 (60%)	43 (61%)	
Yes	53 (40%)	28 (39%)	
Diabetes for more than 10 years			0.093
Not	101 (76%)	61 (86%)	
Yes	32 (24%)	10 (14%)	
Insulin Use			0.4
Not	98 (74%)	56 (79%)	
Yes	35 (26%)	15 (21%)	
Preoperative Retinal Edema			<0.001
Not	109 (82%)	7 (9.9%)	
Yes	24 (18%)	64 (90%)	
Subretinal Fluid			<0.001
Not	114 (86%)	22 (31%)	
Yes	19 (14%)	49 (69%)	
Intact ELM			<0.001
Yes	20 (15%)	28 (39%)	
Not	113 (85%)	43 (61%)	
Intact EZ			<0.001
Yes	43 (32%)	62 (87%)	
Not	90 (68%)	9 (13%)	
Intact RPE			0.2
Yes	23 (17%)	18 (25%)	
Not	110 (83%)	53 (75%)	
Inner Retinal Structure Disorder			<0.001
Not	122 (92%)	29 (41%)	
Yes	11 (8.3%)	42 (59%)	
Outer Retinal Structure Disorder			<0.001
Not	81 (61%)	7 (9.9%)	
Yes	52 (39%)	64 (90%)	
Age	62 (55, 69)	63 (57, 69)	0.8
Preoperative Vision	0.25 (0.15, 0.40)	0.25 (0.12, 0.30)	0.029
Preoperative CRT	327 (288, 385)	492 (444, 577)	<0.001
Total Bilirubin	8.0 (5.5, 11.0)	7.0 (4.6, 9.0)	0.072
Triglycerides	1.27 (0.94, 2.29)	1.55 (1.13, 2.34)	0.3
Total Cholesterol	4.56 (3.62, 5.62)	4.66 (4.23, 5.67)	0.2
High Density Lipoprotein	1.09 (0.95, 1.34)	1.15 (0.90, 1.38)	>0.9
Low Density Lipoprotein	2.67 (1.84, 3.41)	3.03 (2.39, 4.00)	0.031
Apolipoprotein A	1.36 (1.20, 1.49)	1.33 (1.13, 1.58)	>0.9
Fasting Blood Glucose	7.01 (5.85, 10.00)	6.59 (5.06, 9.04)	0.10
Blood Urea	6.50 (5.36, 7.99)	7.20 (5.51, 8.63)	0.021
Serum Creatinine	76 (61, 91)	84 (67, 125)	0.012
Uric Acid	346 (283, 415)	359 (313, 437)	0.2
Calcium	2.38 (2.33, 2.43)	2.33 (2.20, 2.43)	0.005
Magnesium	0.89 (0.84, 0.93)	0.90 (0.81, 0.95)	0.7
White Blood Cell Count	6.70 (5.50, 7.60)	6.80 (5.80, 8.30)	0.2
Neutrophil Count	3.90 (2.80, 4.90)	4.20 (3.30, 5.30)	0.12
Lymphocyte Count	2.10 (1.60, 2.40)	2.00 (1.60, 2.40)	0.8
Red Blood Cell Count	4.45 (3.97, 4.83)	4.28 (3.64, 4.71)	0.025
Hemoglobin	130 (119, 146)	120 (106, 142)	0.029
Platelet Count	223 (197, 272)	248 (203, 302)	0.14
Mean Platelet Volume	10.20 (9.50, 10.80)	9.90 (9.40, 10.40)	0.042
Platelet Distribution Width	11.30 (10.00, 12.80)	10.90 (9.90, 11.70)	0.050
Glycated Hemoglobin concentration	66 (55, 83)	64 (51, 76)	0.2
Glycated Hemoglobin percent	8.20 (7.20, 9.70)	8.20 (6.60, 9.30)	0.2
Glycation	0.40 (0.30, 0.60)	0.40 (0.40, 0.50)	0.7

^a^ n (%); Median (Q1, Q3).

^b^ Pearson’s Chi-squared test; Wilcoxon rank sum test.

In terms of biochemical indicators, patients with central retinal thickness reduction showed significantly higher levels of low-density lipoprotein (*p* = 0.031), blood urea nitrogen (*p* = 0.021), and serum creatinine (*p* = 0.012). Additionally, red blood cell count (*p* = 0.025), hemoglobin (*p* = 0.029), and mean platelet volume (*p* = 0.042) were significantly lower in these patients.

### 3.2 Feature selection

**FIGURE 2:**
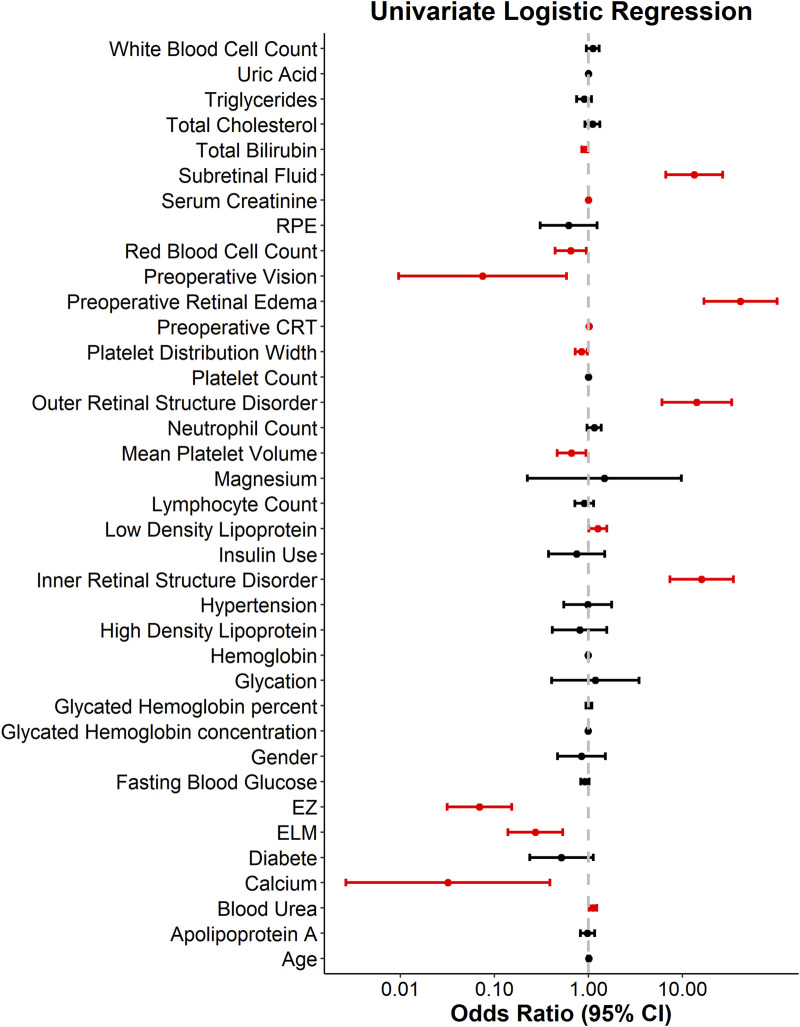
Forest plot of univariate logistic regression for predicting anatomical response to anti-VEGF therapy.

Among the 37 collected variables, a total of 16 predictors were identified as independent factors associated with central retinal thickness reduction following anti-VEGF treatment. Specifically, preoperative visual acuity, intact ELM, intact EZ, total bilirubin, serum calcium, red blood cell count, mean platelet volume, and platelet distribution width were identified as negative predictors (i.e., protective factors). In contrast, preoperative CRT >400 μm, presence of preoperative retinal edema, subretinal fluid, inner retinal structure disorder, outer retinal structure disorder, LDL, blood urea, and serum creatinine were identified as positive predictors of central retinal thickness reduction (Figure 2).

To further control for potential confounding, LASSO regression was applied for refined feature selection. Among the 37 initial candidate variables, five predictors were selected via LASSO: Preoperative CRT >400 μm, Presence of preoperative retinal edema, Presence of subretinal fluid, Integrity of the EZ and Inner retinal structure disorder.

**FIGURE 3:**
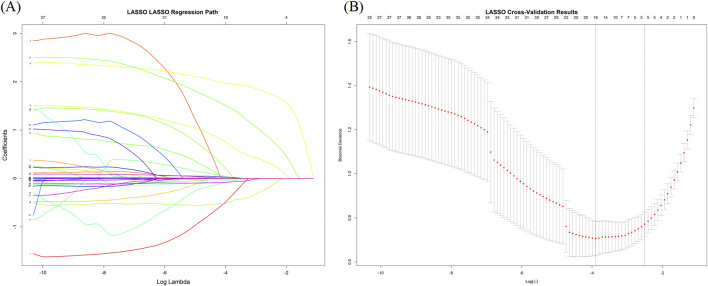
Feature selection using LASSO regression. **(A)** LASSO coefficient profiles of the 37 candidate predictors. **(B)** Ten-fold cross-validation for selecting the optimal lambda in LASSO regression.

Multicollinearity analysis of these five variables showed that all VIF values were below 2.5, indicating no significant multicollinearity among the selected features (Figure 3; Table 2).

**TABLE 2:** Variance inflation factor (VIF) method checks the multicollinearity of the independent variable.

Variable	VIF	OR value for logistic regreesion
Preoperative CRT	2.18	1.014 (1.010,1.019)
Preoperative Retinal Edema	2.46	41.52 (16.93,101.80)
Subretinal Fluid	2.09	13.36 (6.64,26.89)
Intact EZ	1.95	0.069 (0.032,0.15)
Inner Retinal Structure Disorder	1.48	16.06 (7.38,34.96)

### 3.3 Model comparison and evaluation

**FIGURE 4:**
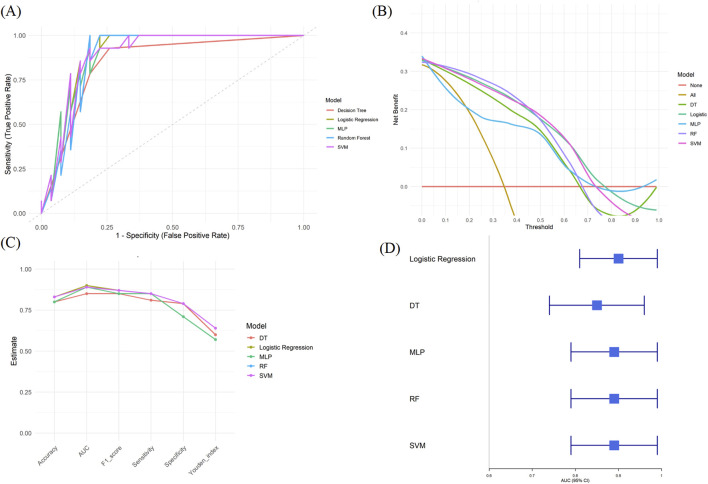
Performance comparison of five machine learning models in predicting anatomical response to anti-VEGF therapy. **(A)** ROC curves of five models **(B)** DCA curves of five models **(C)** Bar plot comparing model performance across six metrics **(D)** Forest plot of AUC values and 95% confidence intervals for each model.

Based on the selected variables, five machine learning models were developed: DT, Logistic Regression, MLP, RF, and SVM. Among these, the Logistic Regression model demonstrated the best performance in the internal validation cohort (Figures 4A, C, D).

The Logistic model achieved an accuracy of 0.83, sensitivity of 0.85, specificity of 0.79, F1-score of 0.87, and AUC of 0.90 (95% CI: 0.81–0.99). The Youden index was 0.64.

The DCA curve showed that across a threshold range of 0–0.78, the logistic model provided greater net clinical benefit compared to “treat all” or “treat none” strategies. Detailed performance metrics for all models are shown in Table 3; Figure 4B.

**TABLE 3:** Different model metrics evaluation of retinal thickness decrease prediction.

Model	Accuracy	AUC(95% Cl)	Sensitivy	Specificity	Youden index	F1 score
Logistic Regression	**0.83**	**0.90(0.81–0.99)**	**0.85**	**0.79**	**0.64**	**0.87**
DT	0.80	0.85 (0.74,0.96)	0.81	0.79	0.60	0.85
MLP	0.80	0.89 (0.79,0.99)	0.85	0.71	0.57	0.85
RF	0.83	0.89 (0.79,0.99)	0.85	0.79	0.64	0.87
SVM	0.83	0.89 (0.79,0.99)	0.85	0.79	0.64	0.87

Note: Bold values highlight the best result observed for each metric across the five predictive models.

### 3.4 Model interpretability and clinical application

**FIGURE 5:**
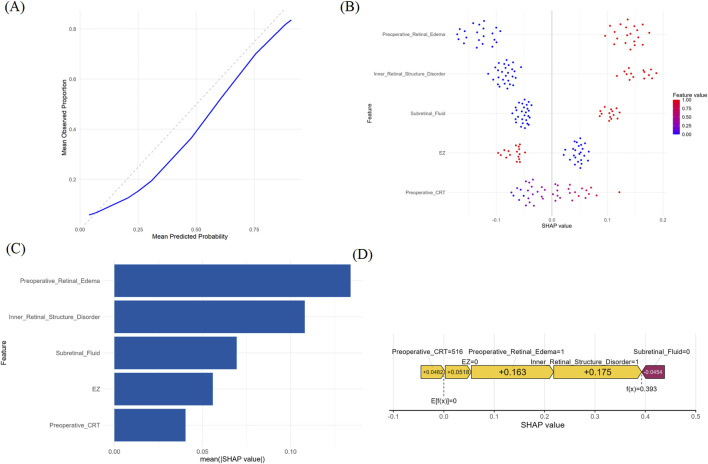
Interpretability and explainability of the optimal prediction model using SHAP analysis. **(A)** Calibration curve of the logistic regression model. **(B)** SHAP summary plot **(C)** SHAP bar plot ranking the average absolute impact of each feature **(D)** SHAP force plot illustrating the individualized prediction explanation for a representative case.

The calibration curve was used to compare predicted probabilities with observed event rates. The calibration curve of the logistic regression model closely followed the ideal diagonal line (perfect calibration), indicating good agreement between predicted and observed outcomes. Although slight deviation was observed in the mid-probability range, the model overall demonstrated satisfactory calibration (Figure 5A).

The SHAP analysis revealed the influence of individual features on model output. In the SHAP summary plot (Figure 5B), each dot represents one patient; colour encodes the raw feature value and the x-axis indicates the marginal contribution (SHAP value) to the log-odds of achieving ≥20% CRT reduction. Pre-operative retinal oedema emerged as the strongest positive driver of treatment response, followed in descending order by the inner retinal layers disorder, presence of subretinal fluid, disruption of the EZ, and baseline CRT (Figure 5C). Importantly, EZ integrity exerted a negative influence—eyes with an intact EZ were less likely to exhibit a large anatomical response (Figure 5C).

To further understand the model’s decision-making process, we selected a representative individual and explained their prediction using a SHAP force plot (Figure 5D). The individual had a preoperative CRT of 516 μm, which contributed +0.0482 to the prediction. EZ disruption contributed +0.0518, retinal edema +0.175, and inner retinal structure disorder +0.163. In contrast, absence of subretinal fluid contributed negatively (−0.0454) to the predicted probability of central retinal thickness reduction.

In a *post hoc* evaluation of potential two-way interactions, we generated pair-wise SHAP-dependence plots for all combinations of the five final predictors (Supplementary Figure S1). In each panel the colour bands remained essentially parallel and no “fan-out” or crossover pattern was observed, indicating that the marginal effect of any predictor was not materially modified by the level of a second predictor.

**FIGURE 6:**
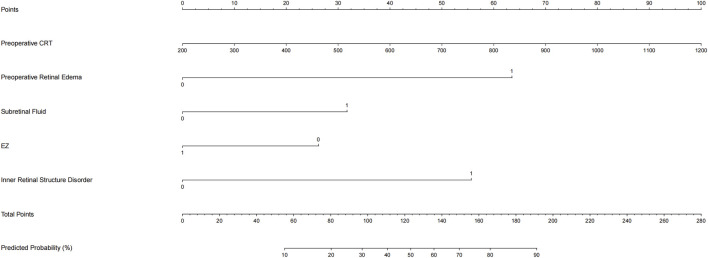
Nomogram for predicting anatomical response to anti-VEGF therapy in DME.

A nomogram was developed based on the logistic regression model to provide clinicians with an intuitive tool for individualized prediction (Figure 6). By translating patient-specific clinical features into point scores, clinicians can calculate a total score and estimate the probability of central retinal thickness reduction after anti-VEGF therapy. The nomogram allows for rapid bedside risk stratification and personalized treatment planning.

## 4 Discussion

Anti-VEGF therapy is currently the first-line treatment for DME. However, its efficacy varies widely among individuals. Predicting treatment response is critical for optimizing therapeutic strategies and achieving personalized care in DME management (Rasti et al., 2020). Previous studies have developed machine learning models using imaging biomarkers alone to predict anti-VEGF treatment outcomes in DME (Allingham et al., 2017; Leng et al., 2024). We expanded on these approaches by incorporating systemic metabolic and hematologic markers, which may account for additional variability in treatment response. As a result, our integrated model achieved robust predictive performance while considering both ocular and systemic factors. For context, prior OCT-only models for DME have reported AUC values around 0.76–0.83 (Gallardo et al., 2021; Shah et al., 2017), whereas our multi-modal model achieved a higher AUC of 0.90 in the present study. Notably, an OCT-based deep learning model recently attained an AUC 0.99 on a small dataset (Song et al., 2025); our approach achieves comparable accuracy while offering greater interpretability. Unlike prior models that functioned as black boxes, the interpretability of our model—achieved through SHAP values and a nomogram—provides clinicians with clear insights into individual risk factors, an advantage that facilitates clinical decision-making. Among various algorithms tested, the logistic regression model demonstrated the best predictive performance and interpretability. Our findings identified several key predictors of post-treatment central retinal thickness reduction, including preoperative retinal edema >400 μm, DRIL, subretinal fluid, EZ integrity, and baseline CRT.

In this study, we compared the performance of five commonly used machine learning algorithms for predicting anatomical response to anti-VEGF therapy in patients with diabetic macular edema. Although nonlinear models such as MLP and RF also demonstrated favorable performance, we ultimately selected the logistic regression model as the preferred approach. This decision was based on its superior overall performance in the internal validation cohort (AUC = 0.90), along with consistently high sensitivity, specificity, and F1 score. Moreover, logistic regression offers a simpler and more interpretable structure, allowing for direct extraction of feature coefficients and facilitating the development of user-friendly clinical tools such as nomograms. Given the relatively limited sample size, logistic regression is also less prone to overfitting and tends to exhibit better generalizability. Therefore, we considered logistic regression to be the most appropriate choice in balancing predictive accuracy with clinical applicability in this context.

The association between baseline CRT and the anatomical response to anti-VEGF therapy has been widely established in previous clinical trials. Studies such as RESTORE and RIDE/RISE have demonstrated that eyes with greater initial CRT experience more substantial reductions in macular thickness and greater visual improvements following ranibizumab treatment (Wells et al., 2016; Pieramici et al., 2016). Real-world data similarly support this finding, with patients having CRT >400 µm showing greater anatomical improvement than those with thinner retinas (Mushtaq et al., 2014). Notably, the UK National Institute for Health and Care Excellence (NICE) guidelines recommend CRT >400 µm as a threshold for initiating anti-VEGF therapy, given the superior efficacy of ranibizumab over laser treatment in this subgroup (Amoaku et al., 2020). Our study aligns with this evidence, suggesting that eyes with more severe edema at baseline (CRT >400 µm) are more likely to exhibit pronounced anatomical responses. This may be attributed to increased VEGF levels and severe blood-retinal barrier disruption in these patients, resulting in greater responsiveness to anti-VEGF agents that effectively neutralize VEGF and reduce vascular leakage (Funatsu et al., 2002; Gurung et al., 2023).Nevertheless, baseline CRT alone cannot fully predict treatment outcomes, which also depend on factors such as baseline visual acuity and OCT morphologic features.

DRIL is an OCT biomarker reflecting the loss of distinct retinal inner layer boundaries within the central 1 mm zone. It is considered a negative prognostic indicator of visual function in DME. Sun et al. first reported that greater baseline DRIL extent was associated with poorer visual acuity, and that early changes in DRIL correlated with subsequent visual outcomes (Das et al., 2018; Sun et al., 2014). Further studies have suggested that DRIL reflects chronic edema- or ischemia-related damage to Müller and bipolar cell axons, potentially hindering rapid resolution of edema after therapy (Munk et al., 2022; Midena et al., 2023; Lai et al., 2023). Our model also indicated that severe baseline DRIL was associated with a lower likelihood of central retinal thickness reduction. We hypothesize that DRIL may reflect underlying neurodegeneration and capillary nonperfusion, limiting the capacity for structural recovery even after VEGF suppression.

Subretinal fluid is a distinct OCT feature in DME, and several studies have shown its presence to be associated with better response to anti-VEGF therapy (Sophie et al., 2015; Hwang et al., 2019; Kwon et al., 2021). For instance, subgroup analyses from the RIDE/RISE trials revealed that patients with baseline subretinal fluid had a higher probability of achieving complete macular dryness (CFT ≤250 µm) after ranibizumab treatment (Sophie et al., 2015). Another prospective study similarly reported that eyes with subretinal fluid exhibited greater CRT reductions and better visual outcomes after anti-VEGF therapy (Huang et al., 2022). Our findings are consistent with these reports and suggest that subretinal fluid is a positive predictor of anatomical improvement. The underlying mechanism may relate to the highly VEGF-driven nature of subretinal fluid-DME, where severe outer blood-retinal barrier disruption leads to fluid accumulation in the subretinal space. In such cases, VEGF blockade can promptly reduce leakage and resolve subretinal fluid (Kwon et al., 2021; Sonoda et al., 2013).

Interestingly, our study found that EZ integrity was a negative predictor of central retinal thickness reduction after anti-VEGF treatment. Although many previous studies have identified intact EZ as a favorable prognostic factor for visual acuity recovery (Hsieh et al., 2023), its role in anatomical improvement is less clear and remains underexplored. EZ continuity generally reflects relatively mild, early-stage disease, with less structural disorganization and lower VEGF levels. Therefore, while these eyes have better preserved photoreceptor function and potential for visual improvement, their baseline macular thickness is often closer to normal, leaving limited room for further reduction. In contrast, disrupted EZ often implies chronic, VEGF-driven damage, greater edema, and worse baseline anatomy, which may respond more dramatically to anti-VEGF therapy in terms of CRT reduction. This inverse relationship has been supported by several real-world studies showing that eyes with greater baseline CRT and structural disruption—often including EZ loss—achieve more pronounced CRT reductions, whereas structurally intact eyes exhibit minimal changes post-treatment (Chaturvedi et al., 2025; Saxena et al., 2022). This suggests that intact EZ may reflect a lower disease burden and lower VEGF expression, making edema less responsive to anti-VEGF therapy in terms of structural reversal. In these cases, although CRT reduction is limited, visual function may still be preserved or improved. Therefore, EZ integrity appears to serve as a dual indicator—predicting better functional outcomes but less dramatic anatomical responses.

To enhance clinical applicability, we transformed the final logistic regression equation into a user-friendly nomogram that allows rapid bedside risk stratification. Clinicians can plot the five baseline predictors—pre-treatment CRT >400 μm, pre-operative retinal edema, presence of subretinal fluid, disorganization of the inner retinal layers, and intactness of the ellipsoid zone—assign the corresponding points, and sum them to obtain a Total Points score. This score can then be vertically projected onto the bottom scale to estimate the probability of achieving a ≥20% reduction in central retinal thickness after anti-VEGF therapy. Internal validation demonstrated excellent calibration and discrimination of the nomogram (AUC = 0.90). In practice, patients with a high predicted probability can proceed with standard anti-VEGF regimens; those with an intermediate probability should be monitored more closely and considered for early adjunctive therapy; and those with a low probability may benefit from prompt evaluation of alternative or combined treatments. The transparency and interpretability of this tool provide an intuitive quantitative basis for individualized management of DME. Moreover, explainable AI methods such as SHAP improve model transparency by revealing how each variable contributes to the prediction, thereby enhancing clinical interpretability and decision-making. Pair-wise SHAP-dependence results indicate that the effects of baseline CRT, retinal oedema, sub-retinal fluid, DRIL and EZ integrity act largely additively in our cohort, implying that the logistic model’s behaviour is dominated by main effects. This additivity not only enhances interpretability but also supports the construction of a straightforward main-effect nomogram for clinical use.

This study has several limitations. First, it was conducted at a single center with a limited sample size, which may introduce selection bias. External validation in larger, multicenter cohorts is needed to improve generalizability. Second, the study focused on anatomical response (≥20% CRT reduction) and did not include visual acuity outcomes or neuro electrophysiological monitoring, which limits the comprehensiveness of treatment assessment (Scott et al., 2024; Kremers and Huchzermeyer, 2024). Third, the follow-up period was relatively short, and the model’s ability to predict long-term outcomes remains untested. Finally, while systemic metabolic variables were included, important biological markers such as VEGF and inflammatory cytokines were not measured. Future studies should incorporate these factors to enhance model performance.

## 5 Conclusion

In this study, we developed and validated a machine learning model with good predictive value for assessing anatomical response to anti-VEGF therapy in patients with DME. Preoperative retinal edema >400 μm, DRIL, presence of subretinal fluid, and EZ integrity were identified as key factors influencing the likelihood of central retinal thickness reduction following treatment. Future research involving larger, multicenter cohorts and incorporation of more comprehensive biological and clinical markers is warranted to further refine and optimize the model.

## Data Availability

The original contributions presented in the study are included in the article/Supplementary Material, further inquiries can be directed to the corresponding authors.

## References

[B1] Allingham M. J. Mukherjee D. Lally E. B. Rabbani H. Mettu P. S. Cousins S. W. (2017). A quantitative approach to predict differential effects of anti-VEGF treatment on diffuse and focal leakage in patients with diabetic macular edema: a pilot study. Transl. Vis. Sci. Technol. 6 (2), 7. 10.1167/tvst.6.2.7 PMC5374879 28377846

[B2] Amoaku W. M. Ghanchi F. Bailey C. Banerjee S. Banerjee S. Downey L. (2020). Diabetic retinopathy and diabetic macular oedema pathways and management: UK Consensus Working Group. Eye 34 (Suppl. 1), 1–51. 10.1038/s41433-020-0961-6 PMC7337227 32504038

[B3] Bellemo V. Lim Z. W. Lim G. Nguyen Q. D. Xie Y. Yip M. Y. T. (2019). Artificial intelligence using deep learning to screen for referable and vision-threatening diabetic retinopathy in Africa: a clinical validation study. Lancet Digit. Health 1 (1), e35–e44. 10.1016/S2589-7500(19)30004-4 33323239

[B4] Chaturvedi S. Paul A. Singh S. Akduman L. Saxena S. (2025). The ellipsoid zone is a structural biomarker for visual outcomes in diabetic macular edema and macular hole management. Vis. (Basel) 9 (1), 4. 10.3390/vision9010004 PMC11755456 39846620

[B5] Das R. Spence G. Hogg R. E. Stevenson M. Chakravarthy U. (2018). Disorganization of inner retina and outer retinal morphology in diabetic macular edema. JAMA Ophthalmol. 136 (2), 202–208. 10.1001/jamaophthalmol.2017.6256 29327033 PMC5838716

[B6] Ehlers J. P. Yeh S. Maguire M. G. Smith J. R. Mruthyunjaya P. Jain N. (2022). Intravitreal pharmacotherapies for diabetic macular edema: a report by the American academy of ophthalmology. Ophthalmology 129 (1), 88–99. 10.1016/j.ophtha.2021.07.009 34446301

[B7] Ensor J. Martin E. C. Riley R. D. (2019). Pmsampsize: calculates the minimum sample size required for developing a multivariable prediction model. R. package version 1 (1), 1.

[B8] Farahat Z. Zrira N. Souissi N. Bennani Y. Bencherif S. Benamar S. (2024). Diabetic retinopathy screening through artificial intelligence algorithms: a systematic review. Surv. Ophthalmol. 69 (5), 707–721. 10.1016/j.survophthal.2024.05.008 38885761

[B9] Funatsu H. Yamashita H. Noma H. Mimura T. Yamashita T. Hori S. (2002). Increased levels of vascular endothelial growth factor and interleukin-6 in the aqueous humor of diabetics with macular edema. Am. J. Ophthalmol. 133 (1), 70–77. 10.1016/s0002-9394(01)01269-7 11755841

[B10] Gallardo M. Munk M. R. Kurmann T. De Zanet S. Mosinska A. Karagoz I. K. (2021). Machine learning can predict anti–VEGF treatment demand in a treat-and-extend regimen for patients with neovascular AMD, DME, and RVO associated macular edema. Ophthalmol. Retina 5 (7), 604–624. 10.1016/j.oret.2021.05.002 33971352

[B11] Gong D. Li W. T. Li X. M. Wan C. Zhou Y. J. Wang S. J. (2024). Development and research status of intelligent ophthalmology in China. Int. J. Ophthalmol. 17 (12), 2308–2315. 10.18240/ijo.2024.12.20 39697896 PMC11589450

[B12] Guo X. Liu H. Chen X. Wang Z. Li S. Lei B. (2023). Comparison of detection rates and time costs of different fundus imaging techniques for retinal neovascularization in optic disc and other area of proliferative diabetic retinopathy. Chin. J. Exp. Ophthalmol., 654–660. 10.3760/cma.j.cn115989-20210916-00518

[B13] Gurung R. L. FitzGerald L. M. Liu E. McComish B. J. Kaidonis G. Ridge B. (2023). Predictive factors for treatment outcomes with intravitreal anti-vascular endothelial growth factor injections in diabetic macular edema in clinical practice. Int. J. Retina Vitr. 9 (1), 23. 10.1186/s40942-023-00453-0 PMC10074667 37016462

[B14] Hatamnejad A. Orr S. Dadak R. Khanani A. Singh R. Choudhry N. (2024). Anti-VEGF and steroid combination therapy relative to anti-VEGF mono therapy for the treatment of refractory DME: a systematic review of efficacy and meta-analysis of safety. Acta Ophthalmol. 102 (3), e204–e214. 10.1111/aos.15724 37365698

[B15] Hsieh T. C. Deng G. H. Chang Y. C. Chang F. L. He M. S. (2023). A real-world study for timely assessing the diabetic macular edema refractory to intravitreal anti-VEGF treatment. Front. Endocrinol. (Lausanne) 14, 1108097. 10.3389/fendo.2023.1108097 37265702 PMC10230025

[B16] Huang Y. T. Chang Y. C. Meng P. P. Lin C. J. Lai C. T. Hsia N. Y. (2022). Optical coherence tomography biomarkers in predicting treatment outcomes of diabetic macular edema after dexamethasone implants. Front. Med. (Lausanne) 9, 852022. 10.3389/fmed.2022.852022 35755055 PMC9218219

[B17] Hussain R. M. Ciulla T. A. (2016). Treatment strategies for refractory diabetic macular edema: switching anti-VEGF treatments, adopting corticosteroid-based treatments, and combination therapy. Expert Opin. Biol. Ther. 16 (3), 365–374. 10.1517/14712598.2016.1131265 26674182

[B18] Hwang H. B. Jee D. Kwon J. W. (2019). Characteristics of diabetic macular edema patients with serous retinal detachment. Med. Baltim. 98 (51), e18333. 10.1097/MD.0000000000018333 PMC6940188 31860985

[B19] Im J. H. B. Jin Y. P. Chow R. Yan P. (2022). Prevalence of diabetic macular edema based on optical coherence tomography in people with diabetes: a systematic review and meta-analysis. Surv. Ophthalmol. 67 (4), 1244–1251. 10.1016/j.survophthal.2022.01.009 35093404

[B20] Kremers J. Huchzermeyer C. (2024). Electroretinographic responses to periodic stimuli in primates and the relevance for visual perception and for clinical studies. Vis. Neurosci. 41, E004. 10.1017/S0952523824000038 39523890 PMC11579838

[B21] Kwon J. W. Kim B. Jee D. Cho Y. K. (2021). Aqueous humor analyses of diabetic macular edema patients with subretinal fluid. Sci. Rep. 11 (1), 20985. 10.1038/s41598-021-00442-z 34697354 PMC8546094

[B22] Lai D. Wu Y. Shao C. Qiu Q. (2023). The role of müller cells in diabetic macular edema. Invest Ophthalmol. Vis. Sci. 64 (10), 8. 10.1167/iovs.64.10.8 PMC10337800 37418272

[B23] Leng X. Shi R. Xu Z. Zhang H. Xu W. Zhu K. (2024). Development and validation of CNN-MLP models for predicting anti-VEGF therapy outcomes in diabetic macular edema. Sci. Rep. 14 (1), 30270. 10.1038/s41598-024-82007-4 39632987 PMC11618618

[B24] Li L. Xiao K. Shang X. Hu W. Yusufu M. Chen R. (2024). Advances in artificial intelligence for meibomian gland evaluation: a comprehensive review. Surv. Ophthalmol. 69 (6), 945–956. 10.1016/j.survophthal.2024.07.005 39025239

[B25] Mao J. Zhang S. Zheng Z. Deng X. Liu C. Chen Y. (2022). Prediction of anti-VEGF efficacy in diabetic macular oedema using intraocular cytokines and macular optical coherence tomography. Acta Ophthalmol. 100 (4), e891–e898. 10.1111/aos.15008 34403203

[B26] Meng Z. Chen Y. Li H. Zhang Y. Yao X. Meng Y. (2024). Machine learning and optical coherence tomography-derived radiomics analysis to predict persistent diabetic macular edema in patients undergoing anti-VEGF intravitreal therapy. J. Transl. Med. 22 (1), 358. 10.1186/s12967-024-05141-7 38627718 PMC11022368

[B27] Midena E. Torresin T. Schiavon S. Danieli L. Polo C. Pilotto E. (2023). The disorganization of retinal inner layers is correlated to müller cells impairment in diabetic macular edema: an imaging and omics study. Int. J. Mol. Sci. 24 (11), 9607. 10.3390/ijms24119607 37298558 PMC10253687

[B28] Munk M. R. Somfai G. M. de Smet M. D. Donati G. Menke M. N. Garweg J. G. (2022). The role of intravitreal corticosteroids in the treatment of DME: predictive OCT biomarkers. Int. J. Mol. Sci. 23 (14), 7585. 10.3390/ijms23147585 35886930 PMC9319632

[B29] Mushtaq B. Crosby N. J. Dimopoulos A. T. Lip P. L. Stavrou P. El-Sherbiny S. (2014). Effect of initial retinal thickness on outcome of intravitreal bevacizumab therapy for diabetic macular edema. Clin. Ophthalmol. 8, 807–812. 10.2147/OPTH.S56624 24812486 PMC4010623

[B30] Nakano E. Ota T. Jingami Y. Nakata I. Hayashi H. Yamashiro K. (2019). Correlation between metamorphopsia and disorganization of the retinal inner layers in eyes with diabetic macular edema. Graefes Arch. Clin. Exp. Ophthalmol. 257 (9), 1873–1878. 10.1007/s00417-019-04393-0 31227899

[B31] Pieramici D. J. Wang P. W. Ding B. Gune S. (2016). Visual and anatomic outcomes in patients with diabetic macular edema with limited initial anatomic response to ranibizumab in RIDE and RISE. Ophthalmology 123 (6), 1345–1350. 10.1016/j.ophtha.2016.02.007 26992841

[B32] Rasti R. Allingham M. J. Mettu P. S. Kavusi S. Govind K. Cousins S. W. (2020). Deep learning-based single-shot prediction of differential effects of anti-VEGF treatment in patients with diabetic macular edema. Biomed. Opt. Express 11 (2), 1139–1152. 10.1364/BOE.379150 32133239 PMC7041458

[B33] Saxena S. Meyer C. H. Akduman L. (2022). External limiting membrane and ellipsoid zone structural integrity in diabetic macular edema. Eur. J. Ophthalmol. 32 (1), 15–16. 10.1177/11206721211026106 34132138

[B34] Scott M. T. W. Yakovleva A. Norcia A. M. (2024). Visual field asymmetries in responses to ON and OFF pathway biasing stimuli. Vis. Neurosci. 41, E007. 10.1017/S095252382400004X 39698978 PMC11730990

[B35] Sen S. Khalid H. Udaya P. Raman R. Rajendram R. ElHousseini Z. (2025). Ultrastructural imaging biomarkers in diabetic macular edema: a major review. Indian J. Ophthalmol. 73 (Suppl. 1), S7–S23. 10.4103/IJO.IJO_878_24 39723865 PMC11834929

[B36] Shah A. R. Yonekawa Y. Todorich B. Van Laere L. Hussain R. Woodward M. A. (2017). Prediction of anti-VEGF response in diabetic macular edema after 1 injection. J. Vitr. Dis. 1 (3), 169–174. 10.1177/2474126416682569 PMC5668872 29104958

[B37] Song T. Zang B. Kong C. Zhang X. Luo H. Wei W. (2025). Construction of a predictive model for the efficacy of anti-VEGF therapy in macular edema patients based on OCT imaging: a retrospective study. Front. Med. (Lausanne) 12, 1505530. 10.3389/fmed.2025.1505530 40177270 PMC11961644

[B38] Sonoda S. Sakamoto T. Shirasawa M. Yamashita T. Otsuka H. Terasaki H. (2013). Correlation between reflectivity of subretinal fluid in OCT images and concentration of intravitreal VEGF in eyes with diabetic macular edema. Invest Ophthalmol. Vis. Sci. 54 (8), 5367–5374. 10.1167/iovs.13-12382 23860753

[B39] Sophie R. Lu N. Campochiaro P. A. (2015). Predictors of functional and anatomic outcomes in patients with diabetic macular edema treated with ranibizumab. Ophthalmology 122 (7), 1395–1401. 10.1016/j.ophtha.2015.02.036 25870079

[B40] Sun J. K. Lin M. M. Lammer J. Prager S. Sarangi R. Silva P. S. (2014). Disorganization of the retinal inner layers as a predictor of visual acuity in eyes with center-involved diabetic macular edema. JAMA Ophthalmol. 132 (11), 1309–1316. 10.1001/jamaophthalmol.2014.2350 25058813

[B41] Waldstein S. M. Vogl W. D. Bogunovic H. Sadeghipour A. Riedl S. Schmidt-Erfurth U. (2020). Characterization of drusen and hyperreflective foci as biomarkers for disease progression in age-related macular degeneration using artificial intelligence in optical coherence tomography. JAMA Ophthalmol. 138 (7), 740–747. 10.1001/jamaophthalmol.2020.1376 32379287 PMC7206537

[B42] Wan C. Cheng J. Yang W. Chen L. (2025). DBMAE-Net: a dual branch multi-scale feature adaptive extraction network for retinal arteriovenous vessel segmentation. Biomed. Signal Process. Control 104, 107619. 10.1016/j.bspc.2025.107619

[B43] Wei J. Xiao K. Cai Q. Lin S. Lin X. Wang Y. (2024). Meibomian gland alterations in allergic conjunctivitis: insights from a novel quantitative analysis algorithm. Front. Cell Dev. Biol. 12, 1518154. 10.3389/fcell.2024.1518154 39834396 PMC11743466

[B44] Wells J. A. Glassman A. R. Jampol L. M. Aiello L. P. Antoszyk A. N. Baker C. W. (2016). Association of baseline visual acuity and retinal thickness with 1-year efficacy of aflibercept, bevacizumab, and ranibizumab for diabetic macular edema. JAMA Ophthalmol. 134 (2), 127–134. 10.1001/jamaophthalmol.2015.4599 26605836 PMC5567793

[B45] Xiao K. Lu W. Zhang X. Lin S. Wei J. Lin X. (2024). An integrative predictive model for orthokeratology lens decentration based on diverse metrics. Front. Med. (Lausanne) 11, 1490525. 10.3389/fmed.2024.1490525 39464268 PMC11502374

[B46] Yuan J. (2019). Guidelines for artificial intelligent diabetic retinopathy screening system based on fundus photography. Chin. J. Exp. Ophthalmol. 37 (8), 593–598. 10.3760/cma.j.issn.2095-0160.2019.08.001

[B47] Zhang J. Zhang J. Zhang C. Zhang J. Gu L. Luo D. (2022). Diabetic macular edema: current understanding, molecular mechanisms and therapeutic implications. Cells 11 (21), 3362. 10.3390/cells11213362 36359761 PMC9655436

[B48] Zhou J. Song S. Zhang Y. Jin K. Ye J. (2022). OCT-based biomarkers are associated with systemic inflammation in patients with treatment-naïve diabetic macular edema. Ophthalmol. Ther. 11 (6), 2153–2167. 10.1007/s40123-022-00576-x 36166152 PMC9587150

