# Supplementary material for: A machine learning model for predicting anatomical response to Anti-VEGF therapy in diabetic macular edema
Source: Front Cell Dev Biol. 2025 May 30;13:1603958. doi: 10.3389/fcell.2025.1603958 (PMC12162914; doi:10.3389/fcell.2025.1603958)
Supplement: Supplementary file 1 [file DataSheet1.docx]

**Supplementary materials**

**Supplementary Table 1. Results of Shapiro–Wilk Normality Tests for Continuous Variables**

| Variables | W value | P value |
| --- | --- | --- |
| Age | 0.9744 | 0.0009 |
| Preoperative Vision | 0.9419 | ＜0.0001 |
| Preoperative CRT | 0.8534 | ＜0.0001 |
| Total Bilirubin | 0.7556 | ＜0.0001 |
| Triglycerides | 0.582 | ＜0.0001 |
| Total Cholesterol | 0.9655 | 0.0001 |
| High Density Lipoprotein | 0.832 | ＜0.0001 |
| Low Density Lipoprotein | 0.0962 | ＜0.0001 |
| Apolipoprotein A | 0.0515 | ＜0.0001 |
| Fasting Blood Glucose | 0.9387 | ＜0.0001 |
| Blood Urea | 0.8648 | ＜0.0001 |
| Serum Creatinine | 0.4528 | ＜0.0001 |
| Uric Acid | 0.9779 | 0.0027 |
| Calcium | 0.0449 | ＜0.0001 |
| Magnesium | 0.6258 | ＜0.0001 |
| White Blood Cell Count | 0.9721 | 0.0004 |
| Neutrophil Count | 0.9226 | ＜0.0001 |
| Lymphocyte Count | 0.3362 | ＜0.0001 |
| Red Blood Cell Count | 0.9667 | 0.0001 |
| Hemoglobin | 0.9576 | ＜0.0001 |
| Platelet Count | 0.9615 | ＜0.0001 |
| Mean Platelet Volume | 0.0662 | ＜0.0001 |
| Platelet Distribution Width | 0.9309 | ＜0.0001 |
| Glycated Hemoglobin concentration | 0.9202 | ＜0.0001 |
| Glycated Hemoglobin percent | 0.405 | ＜0.0001 |
| Glycation | 0.7024 | ＜0.0001 |

Note: A p-value < 0.05 indicates significant deviation from normality.

**Supplementary Figure 1.Pairwise SHAP-dependence plots illustrating potential feature interactions in the logistic-regression model.** (A) Preoperative CRT points color-coded by EZ status, (B) Preoperative CRT; color-coded by DRIL, (C) Preoperative CRT; color-coded by Pre-operative Retinal Edema, (D) Preoperative CRT; color-coded by EZ integrity, (E) Pre-operative Retinal Edema; color-coded by SRF, (F) SRF; color-coded by DRIL, (G) Pre-operative Retinal Edema; color-coded DRIL, (H) SRF; color-coded by EZ, (I) Pre-operative Retinal Edema; color-coded EZ integrity, (J) EZ integrity; color-coded by DRIL.
